# Emerging Perspectives on the Set of Conditions That Lead to the Emergence of Metabolic Syndrome

**DOI:** 10.3390/jpm14010032

**Published:** 2023-12-26

**Authors:** Bogdan M. Tarcău, Laura G. Vicaș, Lorena Filip, Florin Maghiar, Mircea Șandor, Annamaria Pallag, Tunde Jurca, Mariana Eugenia Mureșan, Eleonora Marian

**Affiliations:** 1 Doctoral School of Biomedical Science, University of Oradea, 1 University Street, 410087 Oradea, Romania; tarcau.bogdanmihai@student.uoradea.ro; 2 Department of Pharmacy, Faculty of Medicine and Pharmacy, University of Oradea, 29 Nicolae Jiga Street, 410028 Oradea, Romania; apallag@uoradea.ro (A.P.); tjurca@uoradea.ro (T.J.); emarian@uoradea.ro (E.M.); 3 Department of Bromatology, Hygiene, Nutrition, Faculty of Pharmacy, Iuliu Hațieganu University of Medicine and Pharmacy, 400349 Cluj-Napoca, Romania; lfilip@umfcluj.ro; 4 Medical Department, Faculty of Medicine and Pharmacy, University of Oradea, 10 1st December Square, 410073 Oradea, Romania; fmaghiar@uoradea.ro; 5 Department of Surgical Disciplines, Faculty of Medicine and Pharmacy, University of Oradea, 10 1st December Square, 410073 Oradea, Romania; msandor@uoradea.ro; 6 Department of Preclinical Discipline, Faculty of Medicine and Pharmacy, University of Oradea, 10 1st December Square, 410073 Oradea, Romania; mmuresan@uoradea.ro

**Keywords:** hypertriglyceridemia, hyperglycemia, dyslipidemia, metabolic syndrome, diet interventions

## Abstract

Metabolic syndrome, as a medical condition, presents multifactorial complexity that is characterized by the resulting damage from genetic, environmental, and lifestyle factors (presence or absence of physical activity, food choices). Thus, metabolic syndrome qualifies unequivocally as a medical condition in which there are, simultaneously, several independent metabolic risk factors, namely, abdominal obesity, high triglyceride level, low HDL cholesterol level, arterial hypertension, and high glycemic level. Although age, sex, socio-economic status, and the precise definition of metabolic syndrome all influence the prevalence and risk of developing the condition, clinical and epidemiological studies clearly show that central obesity, as measured by an increased abdominal circumference, is the main risk factor. Thus, due to the growing global incidence of obesity, there has been an increase in the incidence of metabolic syndrome. Starting with obesity, all other metabolic risk factors are influenced: for example, as a result of insulin resistance with hyperglycemia, diabetes is linked to an increased risk of cardiovascular disease due to increased abdominal circumference. Through this review, we aimed to highlight the latest research studies and dietary nutritional interventions useful in the prevention of this disease but also implementation strategies for primary prevention among the healthy population.

## 1. Introduction

The syndrome of metabolic abnormalities is defined as a worldwide public health issue due to the clinical, metabolic, biochemical, and other correlated changes that it implies, alongside the concomitant presence of several pathological conditions, including the following: abdominal adiposity, hypertension, dyslipidemia, and insulin resistance [1,2]. All these changes, which are specific to metabolic syndrome, produce an increase in type II diabetes, heart events, heart attacks, and non-alcoholic hepatic steatosis [3,4,5]. In excess of an impact of lifestyle, the lack or presence of physical activity, and a balanced diet of macronutrients and micronutrients, metabolic syndrome is also affected by one’s predisposition to diseases [2,6,7,8].

**Figure 1:**
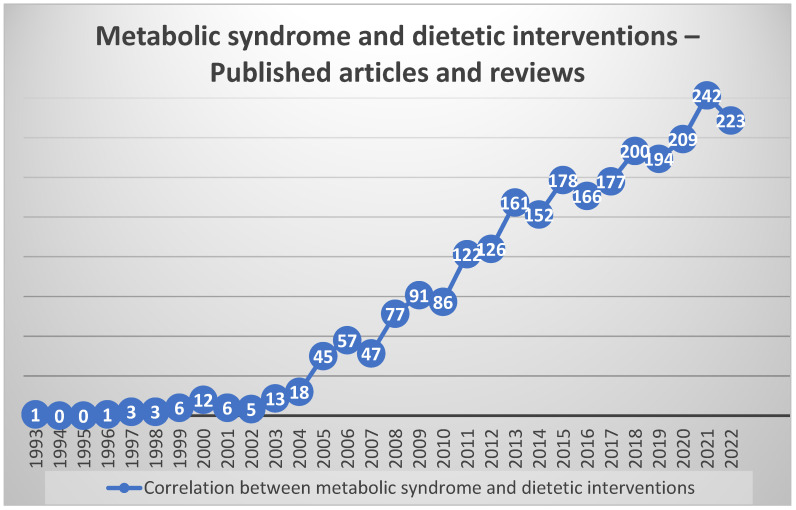
Trends in the quantity of papers and reviews pertaining to metabolic syndrome and dietary therapies across time [9].

The development of publications on metabolic syndrome and dietetic treatments through time is depicted in Figure 1. This is an exciting approach and, as we can see, there has been a steady increase in the research on these topics over the years, making nutritional dietetics a new and significant part of a multidisciplinary medical team.

The rate of occurrence of metabolic syndrome has escalated significantly, posing a substantial public health concern in the United States of America. The frequency of this condition among women increased significantly with age (23.5%) but did so just marginally among males (2.2%) during 1988–1994 and 1999–2000. In 2000, approximately 55 million people in the United States of America were affected by the illness. Mexican American people had a 31.9% age-adjusted prevalence; non-Hispanic white people had a 23.8% prevalence, and African American people had a 21.6% prevalence [10].

Following the investigation of monogenic diseases with characteristics similar to metabolic syndrome, several genes responsible for the appearance of this syndrome among the general population have been determined. Thus, the data provided after a meta-analysis of genome-associated studies determined the common genotypes of single-nucleotide polymorphism in the pathophysiology of metabolic syndrome. Statistically, it is estimated that metabolic syndrome affects nearly 20–25% of the adult population and that this fraction of the population has a 5-fold-increased chance of developing type II diabetes in contrast to individuals without this metabolic condition [8]. According to International Diabetes Federation statistics, sedentary lifestyles, high socio-economic status, and an increased body mass index have been associated with a higher occurrence of metabolic disorders [3]. Along with the factors listed above, it has been shown that the frequency of this metabolic disorder and its constituent elements is influenced by smoking, hereditary predisposition to diabetes, diet, and education [11]. According to the Framingham Heart Study, there is a 45% chance of developing this metabolic syndrome from a weight increase of 2.25 kg over approximately 16 years [12]. The longitudinal design of the Framingham Study has enabled researchers to monitor people over prolonged durations, yielding vital insights into the temporal course of metabolic syndrome and its association with obesity. The results underscore the need to maintain a healthy weight and highlight obesity as a changeable risk factor in the prevention of or reduction in the consequences associated with metabolic syndrome.

## 2. Article Types

**Figure 2:**
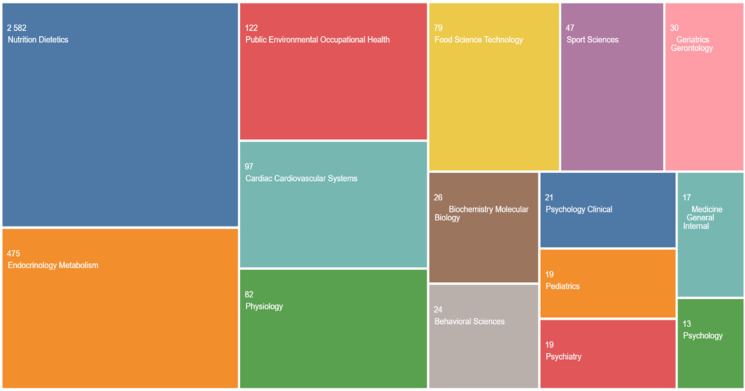
Scientific publications categorized and included in the Scopus database [9].

The current review was based on empirical findings derived from a comprehensive study of metabolic syndrome (MS) literature. This review involved a search of the literature Includes a compilation of published scientific research regarding metabolic syndrome. The chosen papers encompassed both present and earlier works, without any limitations on their publication dates, in order to gather the necessary facts for the research. Furthermore, the Web of Science Core Collection has been most suitable database for the aforementioned goal. Figure 2 depicts the distribution of publications in the Scopus database, scientific publications categorized in the 15 most relevant classifications [9].

**Figure 3:**
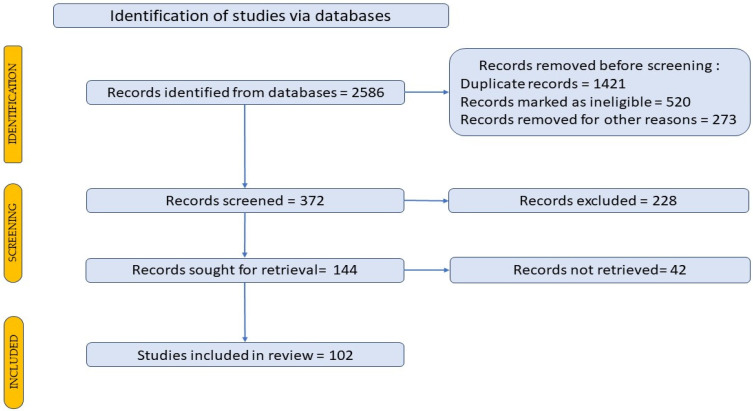
The Prisma flow diagram outlines the selection procedure for bibliographic references.

The written works were retrieved by use conventional keywords for search purposes from scientific journals published between January 2008 and March 2023 to find original data, findings, and scholarly articles within the discipline. The search terms employed to locate articles were as follows: hypertriglyceridemia, hyperglycemia, dyslipidemia, personalized nutrition, metabolic syndrome, nutrigenetics and personalized diet interventions. A grand number of 2582 scientific publications were identified. As illustrated in Figure 3, a Prisma flow diagram has been utilized to demonstrate the way publications were chosen, with the publications included in the review [13,14].

### 2.1. Metabolic Syndrome (MS) Is a Metabolic Condition Defined by Specific Clinical and Paraclinical Criteria

The resistance to insulin is a prominent characteristic of MS, including several cardiovascular risk factors. This syndrome has numerous elements, including Reduced levels of high-density lipoprotein cholesterol (HDL cholesterol), elevated triglyceride levels, high blood pressure, an increased abdominal circumference, and/or a body mass index (BMI) higher than 30 kg/m^2^. As a result, clinical, metabolic, biochemical, and changes take place [7]. This syndrome is defined as a pathological condition in which three or more factors are present: Males with an abdomen circumference exceeding 102 cm and females with an abdominal circumference exceeding 89 cm, blood triglyceride levels exceeding 150 mg/dL, serum HDL cholesterol levels values below 40 mg/dL for men and below 50 mg/dL for women, and high blood pressure (numbers more than 130/85 mmHg) or fasting glucose levels greater than 100 mg/dL [15,16,17,18,19]. Metabolic syndrome (MS) was given a working definition in 1999 via the World Health Organization (WHO), which would later be improved [15]. According to the WHO, MS is characterized additionally to glucose intolerance, as possessing at least two of the particular criteria: impaired tolerance to glucose (IGT), diabetes mellitus (DM), and/or insulin resistance, elevated arterial pressure (140/90 mm Hg), low levels of HDL-C (35 milligrams per deciliter in men and 39 milligrams per deciliter in women) and/or increased blood triglycerides (150 mg/dL), a body mass index (BMI) > 30 kg per square meter and/or waist/hip relation (WHR) > 0.9 in men and >0.85 in women, and/or waist/hip proportion (WHR) > 0.9 in women are two indicators of central obesity and/or microalbuminuria, also known as an albumin/creatine ratio of less than 30 gm/mg or a urine excretion rate of less than 20 gm/min [20].

According to the description provided by the NCEP ATP III, metabolic syndrome is present when an individual meets at least three of the five requirements stated below: blood pressure more than 130/85 mmHg, waist circumference exceeding 40 inches for men and 35 inches for women, elevated fasting triglyceride (TG) levels over 150 mg/dL, low fasting HDL cholesterol levels below 40 mg/dL for males and 50 mg/dL for women, and elevated fasting blood sugar levels surpassing 100 mg/dL [21]. According to the International Diabetes Federation’s (IDF) Worldwide Definition of Metabolic Syndrome, to be diagnosed, an individual must have a BMI of over 30 kg/m^2^ or a waist circumference that exceeds the ethnic threshold. Additionally, they must have at least two of the following: a waist circumference of 102 cm (40 inches) for men or 88 cm (35 inches) for women, triglyceride levels of 150 mg/dL (1.7 mmol/L), and HDL-cholesterol levels of 40 mg/dL (1.03 mmol/L) for men or 50 mg/dL (1.29 mmol/L) for women. Another criterion includes having a blood pressure higher than 130/85 mm Hg, as well as a fasting glucose level of 110 mg/dL (5.6 mmol/L) [3]. Described by the EGIR (1999), once a person has overweight, lipid disorders, impaired fasting glycemia, and hypertension, as described by the AACE criteria, plus a family history of diabetes, hypertension, or cardiovascular disease (CVD), combined with a lack of physical activity, the AACE diagnostic criteria will be satisfied [20]. The criteria for defining metabolic syndrome have had several variations over time, as follows in Table 1:

The clinical and paraclinical findings have determined the close link between obesity the occurrence of metabolic alterations, such as high blood pressure and reduced sensitivity to insulin, decreased glucose tolerance and dyslipidemia. Metabolic syndrome is characterized as a difficult to control spectrum of metabolic disorders associated with heart disease and diabetes type II [22]. Obesity is especially concerning because the rate of obesity in teenagers has climbed from 17% to 30% in the previous 30 years and the risk of adolescents with obesity becoming obese in adulthood is between 55% and 77% [23]. Metabolic syndrome by the occurrence of this phenomenon is attributed to a complex interplay of genetic and environmental variables. Resistance for insulin, dyslipidemia, hypertension, abdominal adiposity, and chronic stress are the underlying factors [15,17].

#### 2.1.1. The Relationship among Abdominal Excess Weight, Metabolic Syndrome, and Heart Disease

Obesity is characterized by a body mass index (BMI) over 30 and is associated with an increased risk of developing high blood pressure, diabetes, dyslipidemia, and metabolic syndrome. Abdominal obesity, represented by an abdominal circumference exceeding 94 in males, 80 in women, is considered to be one of the primary contributors to the onset of insulin resistance and, eventually, the onset of metabolic syndrome [18,24]. The visceral adipose tissue functions as an endocrine organ, secreting a considerable amount of vasoactive chemicals and adipokines and influencing the likelihood of developing or exacerbating metabolic abnormalities [1,25]. For more than five decades, the relationship between the emergence of resistance to insulin and accumulation of abdominal fat tissue tissue and atherosclerosis has been researched and highlighted by a multitude of studies showing both the increased incidence of diabetes and other pathological conditions specific to metabolic syndrome, elevated levels of low-density lipoprotein cholesterol (LDL), decreased levels of high-density lipoprotein cholesterol (HDL), and hypertension (high blood pressure) and high uric acid. Gulbahar and colleagues demonstrated that measuring serum zinc, total cholesterol, fasting insulin, and fasting blood glucose levels is a practical method for monitoring postmenopausal women with high cardiovascular disease (CVD) risk profiles [26]. Abdominal circumference is a prognosticator of cardiovascular illness and diabetes mellitus. Data were also highlighted through a study that included over 160,000 patients. The data provided by measuring the abdominal circumference were more accurate than the BMI alone. The use of abdominal circumference, in particular, was also highlighted in the EPIC study, which included more than 350,000 people and concluded that a 5 cm increase in abdominal circumference while maintaining BMI led to an increased risk of mortality by 1.117 among men and 1.13 among women [27]. Marketou et al. have demonstrated that visceral obesity significantly impacts cardiovascular health starting at an early stage of an individual’s life. The distribution of visceral adipose tissue is not solely determined by body mass index (BMI) and offers supplementary prognostic insights [28]. Obesity is defined by the accumulation of fat cells in the abdominal adipose tissue. Over time, increasing the fat tissue can lead to development of insulin resistance can subsequently result in the establishment of diabetes. Cytokines that promote inflammation are being produced from adipose tissue, which are adiponectin, leptin, tumor necrosis factor, and plasminogen activator inhibitors, has a negative impact on insulin usage. Obesity is linked to cardiovascular disease, non-alcoholic fatty liver disorder and cancer. Overnutrition and lack of physical activity contribute to fat storage in adipose tissue. Usually, as cells increase, more leptin is secreted to signal the brain’s satiety. However, leptin resistance, like insulin resistance, may occur in obese patients, leading to a lack of satiety during eating [25].

#### 2.1.2. Metabolic Syndrome Is Linked to the Proinflammatory Marker C-Reactive Protein, Which Is also Linked to Diabetes Type 2 and Cardiovascular Disease

**Figure 4:**
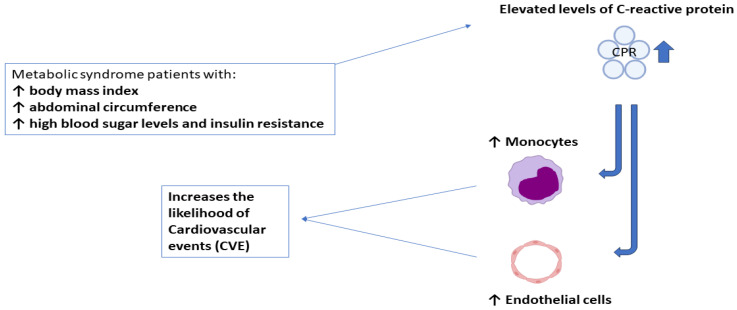
The mechanism of C-reactive protein.

Heightened concentrations of C-reactive protein are directly related to body mass index, abdominal circumference, high blood sugar and resistance to insulin in persons diagnosed with metabolic syndrome [29,30]. Numerous biomarkers of inflammation levels have been investigated to determine their predictive usefulness for CVD (cardiovascular disease). The most prevalent and frequently utilized inflammatory biomarker is C-reactive protein (CRP). Today, several investigations have established that people with MetS (metabolic syndrome) have high CRP levels. High-sensitivity CRP (hsCRP) has also has been proposed as an additional clinical indicator regarding metabolic syndrome and the development of a hsCRP-modified CHD risk [31]. A linear link exists with relation to the quantity of metabolic characteristics and the increasing levels of hsCRP. Rove et al. also found that hs-CRP was negatively linked to the relationship between the amount of HDL cholesterol as well as the sensitivity to insulin index while being positively related to waist circumference, body mass index (BMI), high blood pressure, cholesterol, triglycerides, LDL cholesterol, glucose levels in the plasma and fasting insulin in the “Insulin Resistance and Atherosclerosis Study (IRAS)” [32]. The most vital associations have been discovered between levels of C-reactive protein (CRP), central adiposity, and insulin resistance. Overall, increased CRP in metabolic syndrome patients increases the likelihood of cardiovascular events (CVE) by its impact on vascular cells, especially monocyte activation and the formation of endothelial cell dysfunction. “Low-grade chronic inflammation (CRP level 10 mg/L)” may improve risk prediction. It has been discovered to elevate the likelihood of cardiovascular problems, including metabolic syndrome, on its own [33]. The detailed mechanism of protein C reactive involvement can be better observed schematically in Figure 4:

Recent research looked at the intravascular dynamics of CRP and how it relates to MetS characteristics [34]. The rate of CRP generation, which reflected plasma levels of CRP, was discovered to have a substantial correlation with characteristics of metabolic syndrome (MetS) such as waist circumference, elevated triglyceride levels, low levels of high-density cholesterol, as well as indicators of inflammation and fat tissue biology such as increased levels of interleukin (IL-6) as well as decreased levels of adiponectin. CRP is also produced in adipocytes and can be overexpressed in individuals with central obesity, ultimately leading to insulin resistance and diabetes [34].

#### 2.1.3. Dyslipidemia’s Pathophysiology in the Metabolic Syndrome

**Figure 5:**
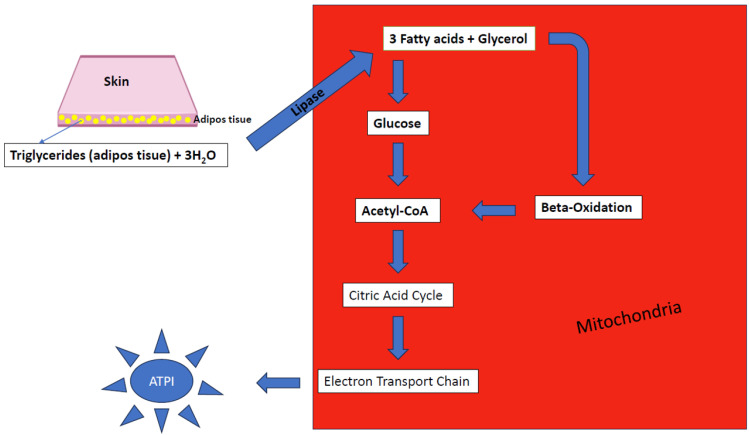
The metabolism of fat.

Triglycerides (TG) are the main form of lipid storage in adipose tissue, stored as spherical inclusions in the cytoplasm of adipocytes [4]. The increased intake of carbohydrates, an intake that exceeds the need adapted to the clinical table, is stored in the body as triglycerides in adipose tissue [35]. The optimal level of triglycerides is shown to be below 150 mg/dL, so any value that exceeds this threshold or is kept below the maximum level with the help of lipid-lowering treatment is regarded as a contributing factor for the development of metabolic syndrome [16]. Starting with glycerol phosphate and fatty acids (GA), the synthesis of TG can take place in three stages. The first stage is when glycerol phosphate can be obtained from a glycolysis intermediate, dihydroxyacetone phosphate. Due to insulin secretion, diets rich in carbohydrates stimulate the synthesis and storage of triglycerides in the form of fat cells. The second one starts when fatty acid levels can be ensured from lipids absorbed in the intestine after ingestion or lipids extracted from adipose tissue [36]. The last one is at the hepatic level, starting from acetyl coenzyme A (acetyl CoA), which can produce a de novo synthesis, the intermediate product comes from the metabolism of amino acids or carbohydrates. Although this triglyceride synthesis can occur in virtually all organs, the Liver and adipose tissue are directly involved. Although the liver is not a storage organ of triglycerides, there is a constant exchange of triglycerides and fatty acids between it and adipose tissue. Triglycerides synthesized in the liver are transported and stored in adipocytes. Lipolysis and lipolysis will cleave adipocytes and triglycerides, and the resulting fatty acids will reach the liver again or be used by catabolism to support the body’s energy needs [36]. The type of fat consumed has a considerable impact on cardiovascular health. Increased consumption of saturated, trans fats raises cholesterol, while monounsaturated and polyunsaturated fats decrease cardiovascular disease risk. HDL cholesterol forms the smallest lipoprotein, containing up to a third of serum cholesterol as cholesterol esters. As a reverse cholesterol process, it transfers excess cholesterol from tissues into the liver or another lipoprotein fractions, such as very low-density lipoprotein cholesterol (VLDL-cholesterol). These peculiarities result in the antiatherogenic role of HDL [36]. Reducing HDL cholesterol levels below 40 mg/dL in males along with 50 mg/dL in females is considered a contributing factor in the occurrence of metabolic syndrome. [4]. A direct link between metabolic disorders, including diabetes, metabolic syndrome, being obese, and low HDL cholesterol, is demonstrated by Girona et al. [37]. The systematic observation of fat metabolism can be seen in Figure 5 below:

#### 2.1.4. The Link between Elevated Fasting Blood Glucose and Risk of Metabolic Syndrome

Measurement of glucose levels in the blood after a period of fasting must have surpassed 100 mg/dL for it to be regarded as an indicator for the onset of metabolic syndrome, or hypoglycemic therapy using oral diabetes medications or insulin must be present [38,39,40].

#### 2.1.5. The Leading Potential Determinant of Developing Metabolic Syndrome Is Hypertension

Hypertension, common among the general populace, is one of the important components related to metabolic disorders. Overall, excessive consumption of fructose and salt can contribute to the development of several factors that contribute to metabolic syndrome, such as insulin resistance, chronic inflammation, activation of the renin angiotensin aldosterone system, increased levels of uric acid in the blood, and obesity. When combined with the increased absorption of salt in the small intestine and renal tubules caused by fructose, these changes will result in excessive salt accumulation and ultimately hypertension [41]. Because obesity contributes to the pathophysiology of metabolic syndrome, hypertension is intimately linked [42]. Hypertension exacerbates cardiovascular cell damage and can ultimately impair the functioning of vital organs such as the kidneys and lungs, which play a crucial role in the progression of cardiovascular disease (CVD) and eventually metabolic syndrome [43]. Because elevated blood pressure, in the context of cardiac disease, plays a crucial role and significantly increases the chances of experiencing a myocardial infarction (MI) and atrial fibrillation, it is regarded as both a disease and a risk factor. High blood pressure, which is also a controllable risk factor, is commonly referred to as a “silent killer”, since it produces harm to the heart and arteries with no obvious symptoms. Elevated blood pressure is a contributing factor for the development of metabolic syndrome: the systolic blood pressure level must be higher than 130 mmHg as well as the diastolic arterial blood pressure must exceed 85 mmHg. In addition to these levels, the presence of hypertension treatment represents a predictive risk for the occurrence of metabolic syndrome. In addition to hypertension, it is being studied if there is no dietary management of the patient, will he later in life develop other cardiometabolic pathologies that ultimately lead to the establishment of metabolic syndrome [44]? More specifically, repeatedly high systolic along with diastolic office blood pressure refers to blood pressure readings that consistently measure 140/90 mmHg or an average home blood pressure reading of 135/85 mmHg [45]. Metabolic syndrome has serious consequences for a person’s health and health care expenses. It is also vital to emphasize the possibility of reducing the incidence of metabolic syndrome due to dietary and lifestyle changes that can bring significant improvements among patients with this disease [46].

### 2.2. Adipogenesis Epigenetic Control in Metabolic Syndrome Development

Epidemiological studies show a close link between intrauterine nutrition, postnatal nutrition, and the increase and onset of metabolic syndrome in adulthood. Mothers who became pregnant in the first two trimesters and were exposed to hunger in the Netherlands from 1944 to 1945 had lower birth weights, but those children were more likely to develop obesity in adulthood. Low-birth-weight children are at the highest likelihood of developing obesity as well as metabolic syndrome during adulthood. This conclusion was also highlighted by the famine to which China was exposed between 1959 and 1961. The cause of obesity and metabolic syndrome can be attributed to the methylation process affecting known as insulin-like growth factor 2 (IGF2) gene and the hypermethylation of two obesity-associated genes, leptin and tumor necrosis factor (TNF) [47]. By methylating DNA in the loci: IGF2-DMR0-A1 and DMR0-A2, a direct relationship was presented between newborns with this methylation and the incidence of obesity in their case [48,49,50]. Animal research revealed that alterations in over/undernutrition and epigenetic modifications in infants influenced growth factors, fatty tissue development, and regulation of appetite. This pattern is assumed to be present in developing countries adults that have a greater prevalence of being obese and having metabolic syndrome [51]. The condition known as hepatic steatosis constitutes a common chronic liver ailment that, in rare situations, can progress to more severe NAFLD stages or the onset of potentially deadly secondary conditions. Conditions affecting the liver’s function itself with examples of conditions that can be affected include non-alcoholic steatohepatitis (NASH), fibrosis, cirrhosis, and hepatocellular cancer. Additionally, other organs such as the heart and blood vessels may also be impacted or the islets of Langerhans (type 2 diabetes) [52].

**Figure 6:**
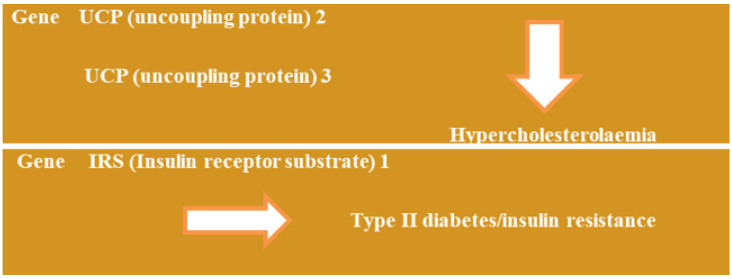
Genetic factors and the risk of altering the clinical parameters [54,55].

The metabolic dysfunction-associated fatty liver illness (MAFLD) exhibits several aspects of the pathophysiology observed in metabolic syndrome due to overweight and obesity, high cholesterol levels, resistance to insulin, mitochondrial damage, an oxidative stress reaction and the production of inflammatory cytokines [53]. The involvement of UCP 2, UCP 3, and IRS 1 genes in metabolic disorders can be observed below in Figure 6:

### 2.3. The Topic of Interest Is the Relationship between Metabolic Syndrome and Non-Alcoholic Fatty Liver Disease

Non-alcoholic fatty liver disease, also referred to as (NAFLD) is the prevailing liver disease globally and the primary cause of liver-related illness and death. By analyzing and evaluating the historical patterns of its global prevalence and incidence, we sought to forecast the burden of NAFLD [56]. The inflammatory form of non-alcoholic fatty liver disease (NAFLD), known as non-alcoholic steatohepatitis (NASH), is connected to the illness’s progression, cirrhosis, and the requirement for a liver transplant. Despite its significance, NASH is not widely understood in clinical practice [57,58]. Over the next decade, the occurrence of “non-alcoholic steatohepatitis” (NASH) is expected to rise by up to 56%. This increase is attributed to the strong association between NASH and the escalating global obesity rates. Hepatocellular carcinoma (HCC) is more commonly attributed to non-alcoholic fatty liver disease (NAFLD) than any other condition in the United States, France, and the United Kingdom. The incidence of hepatocellular carcinoma (HCC) associated with non-alcoholic fatty liver disease (NAFLD) is expected to increase worldwide in parallel with the escalating obesity epidemic [59]. Increased oxidative stress results from an inappropriate equilibrium between synthesizing and removing intracellular reactive oxygen species. As a result, irreparable damage is done to proteins, lipids, DNA, and RNA, which causes molecular changes that alter normal function. Recent research has shown that changed lipids and OSEs are engaged in several phases of the spectrum of “non-alcoholic fatty liver disease (NAFLD)” including fibrosis, hepatocellular cancer, and inflammatory “non-alcoholic steatohepatitis (NASH)” [60,61]. An estimated 3% to 6% of Americans are thought to have NASH, and the prevalence is rising. Being obese, lipid disorders, diabetes type 2, and metabolic syndrome are health conditions that are all closely related to NASH [62,63]. The only approved way to diagnose NASH is by liver biopsy, even though several non-invasive tests and scoring systems identify NAFLD and NASH [64]. NASH is anticipated to overtake other liver diseases as the primary reason for liver transplants in the US, with an estimated 20% of people developing cirrhosis. The mortality rate for individuals with this inflammatory subtype of NAFLD is significantly greater compared to the overall population or patients without this subtype. Specifically, it has an annual all-cause fatality rate of 25.56 per 1000 person years as well as a liver-specific fatality rate of 11.77 per 1000 person years [57].

A fatty liver plays a vital role in a recently discovered connection between liver and pancreatic cells, as well as an increase in glucagon levels, both of which contribute to diabetes pathogenesis [65].

#### Changes in the Diet Plan That Are Imperatively Necessary for “Non-Alcoholic Fatty Liver Disease”

The cornerstone of therapy is lifestyle modification, which includes food adjustments and exercise, with weight loss as the primary objective. Weight reduction is associated with significant improvement in histologic outcomes, including fibrosis. To treat NAFLD, lifestyle adjustments like a low-calorie Mediterranean-style diet and exercising are essential [66]. Some macronutrients that are detrimental for the liver comprise saturated fatty acids (SFA), trans fats, carbohydrates that are simple, and animal proteins. Consuming monounsaturated fats (MUFAs), fatty acids that are polyunsaturated (PUFAs), and omega-3 fatty acids, proteins derived from plants, and dietary fiber is considered to help the liver [67]. As is this review, in contrast to the control group, which followed a World Health Organization-recommended healthy diet, the low-glycemic Mediterranean diet (MedDiet) was typically not energy restricted by the WHO. The MedDiet, which has a low glycemic index, was found to result in a greater decrease in the NAS, as evaluated through the use of ultrasonography after six months of nutritional therapy than the control diet [68]. The Mediterranean-type diet is a popular eating pattern based on the historic dietary practices of Mediterranean Sea nations. It is distinguished by a focus on whole, minimally processed foods, and it comprises the following important components: fruits and vegetables: The Mediterranean diet contains a wide variety of colorful fruits and vegetables. These include important vitamins, minerals, antioxidants, and fiber. Whole Grains: Whole grains, such as whole wheat, brown rice, and oats, are important components of the Mediterranean diet. They contain complex carbs and fiber, which assist in digestion and contribute to a sensation of fullness. Healthy Fats: Olive oil is a staple of the Mediterranean diet. It is high in monounsaturated fats, which are beneficial to the heart. Healthy fats can also be found in nuts, seeds, and avocados. Fish and seafood are rich in omega-3 fatty acids, particularly in fatty fish such as salmon, sardines, and mackerel. In this diet, seafood is a major source of protein. Dairy Consumption is Moderate: Dairy products such as yogurt and cheese are consumed in moderation. These include calcium and protein. Red Meat in Moderation: Red meat is taken in moderation in the Mediterranean diet. Instead, lean protein sources such as chicken and legumes (beans, lentils, and peas) are emphasized. Moderate Alcohol Consumption: Red wine is a typical feature of the Mediterranean diet when used in moderation. It is typically drunk with meals and is thought to offer certain health advantages, owing to its high antioxidant content. Herbs and spices: Instead of salt, fresh herbs and spices are used to flavor foods. Regular physical exercise, while not a dietary component, is an important part of the Mediterranean way of life and adds to general health. Social and Community Interaction: The Mediterranean diet emphasizes the value of social relationships and sharing meals with family and friends, in addition to the food itself. It is worth noting that portion management and mindful eating are also significant components of the Mediterranean diet, which contribute to its health advantages. It also promotes an active lifestyle and a strong feeling of community, both of which are beneficial to general well-being. Nonalcoholic fatty liver disease individuals typically consume a Western-style diet that is high in soda, frozen fast Food, juice, beef and pork fat, processed animal products, whole-fat dairy products, fatty snacks, takeout food, cakes, and cookies while low in cereal, whole-grain foods, vegetables, fruits, extra virgin olive oil, as well as fish [69,70]. Even while losing weight and getting active remain the cornerstones of NAFLD treatment, modifying the diet’s composition regardless of restricting calories may be a viable and long-term approach for NAFLD therapy. The potential therapeutic effect of a “high-quality, wholesome diet” for treating liver steatosis and metabolic disorders in patients with NAFLD without restricting calories or weight loss is presented in this review study [71].

The study affirms that the Mediterranean diet has significant benefits in managing major chronic diseases such as cardiovascular disease, obesity, diabetes, and some types of cancer, based on its fundamental characteristics. Recently, there has been increasing recognition that the Mediterranean Diet, when combined with fitness along with cognitive behavior therapy, may serve as the optimal nutritional plan for preventing and treating NAFLD patients [72,73]. Moreover, the outcomes of multiple nutritional therapy studies have confirmed the advantageous impacts of the Mediterranean diet on non-alcoholic fatty liver disease (NAFLD). In a pilot crossover research including 12 obese individuals with biopsy-confirmed non-alcoholic fatty liver disease (NAFLD), a six-week dietary intervention using the Mediterranean diet (MedDiet) successfully decreased hepatic steatosis (as assessed by NMR) without causing any changes in body weight or waist circumference [74]. Treatment for NAFLD/NASH includes dietary adjustments, weight loss-promoting activity and medications such as vitamin E [75]. Bariatric surgery may be necessary in certain situations to attain and sustain the necessary degree of weight loss for therapeutic effectiveness [57]. Obese/Metabolic NAFLD, which is a kind of NAFLD, is associated with an increased risk of developing type 2 diabetes and cardiovascular disease [76]. The paper “Non-Alcoholic Fatty Liver Disease: Metabolic, Genetic, Epigenetic and Environmental Risk Factors” bases its central hypothesis on the idea that several interrelated factors, such as that the development of non-alcoholic fatty liver disease (NAFLD) in individuals with a genetic susceptibility is influenced by various factors such as insulin resistance, dietary factors, gut microbiota, and genetic and epigenetic factors [77]. Having a comprehensive understanding of the entire range of NAFLD, which includes conditions from obesity through metabolic syndrome and diabetes, might be beneficial in identifying it early and applying personalized treatment [65].

### 2.4. The Function of Nutrigenetics and Nutrigenetics and Its Focus on Nutritional Dietetic Interventions

#### 2.4.1. Exploring the Role of Nutrition, Obesity and Inflammation

Obesity has been associated with persistent, low-level inflammation. When macrophages and T lymphocytes infiltrate adipose tissue, they produce inflammatory chemicals such tumor necrosis factor (TNF), an inhibitor of the plasminogen activation-1 (PAI-1), interleukin-6, interleukin-8, and inflammatory regulators like leptin, resistin, and adiponectin [78]. It is crucial to understand that chronic inflammation is not a distinct illness but rather a mechanistic procedure. Chronic inflammation is linked to various disorders, such as cardiovascular disease (CVD), diabetes, cancer, autoimmune disease, chronic liver disease, and chronic kidney disease [79]. Many areas of metabolism are affected by chronic inflammation, including resistance to insulin, homeostasis of glucose, and lipid metabolism. Environmental variables influencing inflammatory metabolism are connected to food consumption and nutritional status [80]. Western diets heavy in fats that are saturated, sugar, and refined carbohydrates have been related to cardiovascular disease, type 2 diabetes (T2D), obesity, and other metabolic disorders. The Western diet can also enhance the expression of pro-inflammatory cytokines and activate nuclear factor kappa B (NF-kB) after a meal [81]. The Mediterranean diet (MedDiet), on the other hand, is high in monounsaturated fatty acids (MUFA) as well as polyphenols [82]. According to a study by Esmaillzadeh, a diet comparable to the Mediterranean diet decreased the levels of plasma protein C reactive (CRP) and soluble vascular cell adhesion molecule-1 (sVCAM-1), while raising the levels of plasma amyloid A (SAA) and interleukin-6 (IL-6). The results indicate that dietary patterns are associated with increased levels of pro-inflammatory biomarkers in the bloodstream. [83]. Over and above the gold-standard population-based therapies, weight control strategies, and nutrigenomics-guided interventions can support long-term changes in dietary fat intake [84].

#### 2.4.2. The Mediterranean Diet Interacts with Gene Polymorphisms That Are Linked to Atherosclerosis and Inflammation

The MedDiet is distinguished by a high consumption of omega-3 vegetable-based oil, fruits and vegetables, legumes, whole-grained cereals, nuts, seeds, and legumes. Assume moderate alcohol and meat intake, as well as a reduction in dairy, coffee, and red meat consumption. This diet involves antiinflammatory and immune-modulating activities, which reduces pro-inflammatory chemicals such as interleukin (IL-6, IL-8, IL-18), TNF-alpha, and protein C-reactive [85]. It is critical to evaluate the content and quantity of polyphenols within cooking oil, as well as their bioavailability and rate of metabolization by human bodies. The amount of olive oil used in the MedDiet varied between 30 and 50 g/zi, corresponding to a polyphenol consumption of 4 to 9 mg/zi [86]. A recent clinical study assessed the effects of both eicosapentaenoic acid (EPA) and docosahexaenoic acid (DHA) on inflammatory biomarkers in persons with abdominal obesity and subclinical inflammation. During a two-week period, the participants were given supplements in the form of capsules containing either EPA (2.7 g/day), DHA (2.7 g/day), or maize oil (3 g/day; control). Supplements containing EPA and DHA shown superior efficacy compared to maize oil in mitigating inflammation. With all of them, DHA was more effective than EPA at modifying inflammatory biomarkers. In this way, DHA caused a more notable decrease in IL-18 serum levels and a more important increase in adiponectin compared to EPA. Additionally, DHA has decreased plasma levels of CRP, IL-6, IL-18, and TNF-alpha [87].

#### 2.4.3. Managing Cardiometabolic Disease Risk through Diet Interventions

As is well known, the primary diseases with a significant statistical rise closely linked to dietary habits and have been shown to affect gene expression are obesity, type II diabetes, metabolic syndrome, chronic renal insufficiency, and cardiovascular diseases. Because of the complex connections between hereditary and environmental variables, some illnesses are multifactorial. Researchers mention deficiencies in vitamins and minerals caused by insufficient food intake as one of the environmental causes [51]. Another among these genes is TNF alpha, which is involved in inflammatory processes connected to resistance to insulin, obesity, dyslipidemia and cardiovascular disease. The level of ARN measuring TNF alpha levels within the fat tissue as well as skeletal muscle is closely related to fat, patients’ body mass indices (BMI) and inversely correlated with lipoprotein lipase activity [88]. Another gene involved in inflammation and immune system regulation is IL 6, which is released at adiposity levels and significantly affects basal metabolism [89]. Numerous studies have emphasized how diet can affect the activation of hereditary risks for developing diseases like obesity, cardiovascular illness and diabetes type 2. As a result, current data within the domain of public health awareness propose the use of personalized nutritional dietetic plans that are adjusted based on the individual risks of each patient [90]. Patients who exhibit overgrowth or obesity are more likely to have genetic variations affecting fatty acid metabolism, the inflammatory response, and the foam cells. For instance, obesity is directly or indirectly linked to more than 600 nuclear polymorphisms [91].

#### 2.4.4. Exploring the Effect of Diet on Genetic Predisposition

The possibility of developing cardiometabolic diseases is increased when various dietary components are consumed. It has been shown that an eating behavior in which intake of micronutrients, macronutrients, and all bioactive components by the individual needs to be determined through genetic testing results in a reduction in risk of death and an increase in lifetime resulting from decreased susceptibility to cardiometabolic illness. Cardiovascular disease is one of the prevalent diseases, and dietary interventions that increased the intake of fiber, potassium, and folate were associated with decreased mortality [51]. From a pharmaceutical standpoint, taking supplements containing polyphenols can directly inhibit the activity of antioxidants by inhibiting enzymatic activity or indirectly do so. Polyphenols may have antiinflammatory properties and antiproliferative effects [51]. From a nutritional standpoint, both the Dietary Approaches to Stop Hypertension (DASH) and the OmniHeart diet base their recommendations on reducing saturated fat intake and increasing fiber, magnesium, and potassium intake from fruits and legumes. These changes minimize arterial tension and triglyceride levels, which lowers the risk of developing cardiometabolic disorders [92]. In this study, Vajdi et al. provided evidence that maintaining a healthy lifestyle was associated with reduced likelihood of hyperglycemia and high triglyceride levels in Iranian adults [93].

The consumption of Western-type calorically rich diets combined with chronic overnutrition and a sedentary lifestyle in Western societies evokes a state of chronic metabolic inflammation [94]. The lifestyle-modification intervention shows efficacy in resolving metabolic syndrome and reducing the severity of associated abnormalities, including fasting blood glucose, waist circumference, systolic blood pressure, diastolic blood pressure and triglyceride levels, in individuals with metabolic syndrome [95].

On a worldwide scale, these are the major causes of death, although they can be prevented by altering eating habits and lifestyle. From a genetic standpoint, several polymorphisms may predispose a patient to myocardial infarction, arterial hypertension, or diabetes type 2. Even though the exact mechanism is still unclear, it has been shown that patients with elevated levels of apolipoprotein E4 also have elevated LDL cholesterol levels. From a lifestyle perspective, obesity and inactivity can increase the risk of cardiovascular illness [96]. Nutrigenetics seeks to improve the general standard of life by dietary changes that minimize or prevent illness development identified through genetic testing. This enables the use of customized diet plans. Genetic testing is used to identify genetically transmitted diseases such as hypertension, diabetes type 2 and hypercholesterolemia. In the chance of hypertension, the results of the tests are also used to determine how much sodium should be consumed. If SORT1 is present, there is a risk of a plasmatic elevation of LDL cholesterol or total cholesterol [97]. It is important to emphasize, however, that while genetics can contribute to the development of metabolic syndrome, although it is not the only factor involved. Diet, physical exercise, environmental factors, and lifestyle choices all play a significant influence. Insufficient physical activity and an unhealthy diet can result in the development of insulin resistance and obesity, which are key components of metabolic syndrome [18]. Insulin-like growth factor 1 (IGF-1) is a hormone involved in the regulation of growth and development. The liver’s cells synthesize it and subsequently release it into the bloodstream, where it exerts its effects on cells throughout the entire body. IGF-1 has several effects on the body, including promoting the growth and division of cells and regulating the metabolism of carbohydrates and fats [18]. There is some evidence that IGF-1 may contribute to the onset of metabolic syndrome. For example, studies have shown that people with high levels of IGF-1 are more likely to develop resistance to insulin and overweight or obesity, both of which are hallmarks of metabolic syndrome. Nevertheless, the precise correlation between IGF-1 and metabolic syndrome remains incompletely comprehended, necessitating further investigation to validate this association [18].

### 2.5. The Influence of Lifestyle on Metabolic Syndrome

Weight loss is one of the leading therapies that can benefit all MetS disorders. Metabolic syndrome, characterized by overweight or obesity, is primarily caused by an imbalance between energy intake and expenditure. The issue affects nearly 60% of subjects, and 20% of deaths are attributed to poor diet. The following dietary changes should be part of MetS management [36]: consuming fewer saturated and trans fatty acids, which are contained in processed foods, such as commercially baked goods and specific hydrogenated oils, are examples of highly processed food products (the advantages include lower levels of triglycerides and higher HDL-C levels). Incorporating fiber-rich foods, including fruits, vegetables, legumes, and whole grains, into one’s diet will effectively reduce triglyceride levels, boost HDL-C levels, and enhance blood pressure regulation, body weight, and blood sugar. Additionally, vegetables are an excellent potassium source, which helps regulate blood pressure. Increasing omega-3 fatty acids by, for example, seafood consumption (benefits may include lowering the levels of triglycerides). Lowering the amount of dietary carbs (particularly refined ones) to less then 50% of total calorie consumption, mainly by cutting back on sugar-sweetened beverages (benefits include lowering triglyceride levels); and cutting back on sodium intake (the benefits include decreasing blood pressure) [36].

#### 2.5.1. Dietary Recommendations with a Beneficial Effect on the Metabolic Syndrome

Patient lifestyle is related to an essential factor in controlling risk factors for metabolic syndrome. In terms of input food, the replacement of saturated and trans fatty acids from monounsaturated and polyunsaturated fatty acids is an essential non-pharmacological therapeutic means for the control of serum HDL cholesterol, total cholesterol and LDL cholesterol. The American Academy of Pediatrics highlights another critical dietary intervention; the American Association of Cardiology and the World Health Organization refer to the introduction into the diet of vegetables and fruits; these have roles in addition to micronutrients and significant quantities of fiber [1,98]. The outcomes of numerous primary prevention studies have attested to the importance of correct diagnosis and early management of dyslipidemias aimed at reducing cardiovascular mortality and overall mortality. Regarding lifestyle, given the other branches of metabolic syndrome, we can refer to weight loss for lowering abdominal circumference and decreasing the risk of developing insulin resistance. To prevent insulin resistance and type II diabetes, we must also intervene by bringing complex carbohydrates into the diet for a low and moderate glycemic load. Through these interventions, the impact is to lower the risk of hypertension and high fasting blood sugar [98]. To manage metabolic syndrome, it is essential to evaluate the effect of changes in eating behavior by optimizing the lifestyle; this is a mandatory condition in treating metabolic syndrome. This category includes diet, increased physical activity, weight, stopping drinking and alcohol smoking reduction, and emotional and psychological stability [99]. Another one is the pharmacotherapy of dyslipidemias with the help of antihyperlipidemic agents from classes of drugs: statins, fibrates, nicotinic acid, resins, inhibitors, intestinal absorption of cholesterol, omega-3 and fatty acids [4]. It is a significant factor in encouraging and supporting the patient to adhere to the default treatment, especially in cases where compliance may be low, there is concern about secondary effects, and there is a relatively high cost of medication, ignoring or misunderstanding the entire risk of non-treatment [4]. It refers to monitoring lipid fractions, taking measurements of blood pressure, belly circumference, as well as fasting blood glucose at the beginning of the treatment and again 4–6 weeks after starting the medication, and 3–4 months after average values are reached; this is the stage of maintaining the treatment. Along with monitoring them, it is essential to monitor the parameters that may be influenced by therapy (transaminases, creatine kinase) or may control adverse treatment (serum creatinine) [35]. The evaluation refers to the achievements due to compliance and follow-up treatment, reducing the risk of mortality. Compliance with this treatment strategy is essential to control branch levels of metabolic syndrome. Lifestyle optimization is a significant way to manage dyslipidemias, especially if pharmacological treatment is recommended only for patients at risk of moderately increased and increased cardiovascular. By optimizing our lifestyle, we try to lose weight and change body composition by decreasing adipose and visceral tissue, with these changes having positive effects in the control of dyslipidemias. To succeed, lifestyle optimization requires the following: an antihyperlipidemic diet, exercise, weight loss, a reduction in alcohol consumption, smoking cessation and psychosocial optimization [100,101]. Many chronic diseases in a person’s life are directly related to poor food intake. Thus, due to the changes in food that have taken place in recent decades due to industrialization, selection of foods has been directed towards escalated intake of energy-dense foods and heightened levels of saturated fatty acids. In addition to these changes, a decrease in physical activity among the population, resulting in excess calories, led to the onset of obesity and increased risk of metabolic syndrome, along with all its complications (obesity, diabetes, disease cardiovascular disease) [98,101]. The manner in which the selection of various globally recognized diets leads to the improvement of the patient's condition may be observed in detail in Table 2.

#### 2.5.2. The Antihypertensive Diet—The “Dietary Approaches to Stop Hypertension” (DASH) Diet

The DASH diet refers to decreased energy intake and reduced saturated fatty acids and salt information. The proportions of a diet are also found in a healthy person’s case: carbohydrates 55–60%, protein 10–20%, and lipids should not exceed 30% of caloric intake and consist of less than 7% saturated fatty acids [99]. The main characteristic is limited sodium (salt) consumption, crucial for managing blood pressure. This is possible by reducing the amount of processed and packaged foods, as they tend to be high in sodium. Instead, flavoring using herbs, spices, and other seasonings is recommended. An antihypertensive diet, also known as the DASH, is a dietary pattern designed to help lower high blood pressure. It emphasizes nutrient-rich foods and promotes a balanced eating plan. The first step is to incorporate a wide range of vegetables and fruits into your regular diet. These meals are high in fiber, magnesium, and potassium, all of which help to regulate blood pressure. A minimum of 4–5 servings of veggies and fruit are recommended daily. The second step is to replace refined grains with whole grain choices, like bread crafted from whole wheat flour, brown rice, oats, whole wheat pasta, and quinoa. Whole grains provide more fiber and nutrients that support heart health. Protein-rich meals that have a low fat content and a high protein content include without-skin meat, seafood, legumes (beans, lentils), and nuts. They are low in fats that are saturated and contain important nutrients without raising blood pressure. Low-fat or fat-free dairy items such as skimmed milk, yogurt with minimal fat, and reduced-fat cheese, are advised as protein sources. These give important nutrients such as calcium and potassium while keeping saturated fat intake in check [51]. The direct relation between dietary fat intake and the incidence of high cholesterol levels with an increased cardiovascular mortality rate in the “Seven Countries study” is highlighted. This study shows that countries that consume high amounts of saturated fats have higher cholesterol values than countries where carbohydrate consumption predominates complex carbon [102]. In some cases, such as the Tufts trial and the A-Z Weight Loss Study, they have evaluated four types of diets, namely: Atkins (carbohydrate restriction), Ornish (limitation of saturated acids, Weight Watchers or Lears study), hypocaloric) and the Zone study (a diet balanced in macronutrients). The results of the Tufts trial were presented as a short-term reduction with about 10% of the LDL cholesterol to HDL cholesterol; Diet Atkins and A-Z Weight Loss Study showed a decrease of approximately 29% in triglyceride levels and a minor increase in HDL cholesterol (~5%). Research in this area has emphasized the significance of changing the ingested fatty acids (transition from saturated or trans fatty acids to mono- and polyunsaturated) because the increased consumption of saturated fatty acids such as acid lauric, palmitic, and stearic has been linked to an increase in total cholesterol levels and death rate due to cardiovascular disease [27]. The chosen diet must be low in calories because, without this condition, the decrease in triglyceride or LDL cholesterol levels will be nearly insignificant [99].

#### 2.5.3. The Importance of Integrating Foods with a Low Glycemic Index in Daily Diet

Current data demonstrate a correlation between increased glycemic index levels of food and plasma levels of lipid fractions. In this sense, sources of carbohydrates with a moderate glycemic index are recommended, such as green leafy vegetables (broccoli and spinach) and fruits. Thus, replacing the amount of carbohydrates with oily fruits such as nuts or olive oil (rich in monounsaturated fatty acids) significantly reduces hyperglycemia and hypertriglyceridemia, resulting in increased HDL cholesterol and decreased oxidative stress [103]. A study confirmed that a carbohydrate and fat-rich diet, including fructose and fats that are saturated, over a long period elevates the likelihood of developing metabolic syndrome. This leads to abnormal changes in various organs (liver, pancreas, and kidneys) and tissues. These changes occur because of the increased generation of species of reactive oxygen. Following this study, it turned out that the cessation of such a diet will result in a natural recovery of the affected organs and tissues. Patients’ health improved with the changes in diet, which have decreased the possibility of developing metabolic syndrome, reducing mortality [103].

#### 2.5.4. The Significance of Eating Foods High in Fiber and Omega 3

Diets of this type are recommended for non-pharmacological treatment of dyslipidemias because they help reduce triglyceride plasma levels [75]. A meta-analysis found that thirteen cohort studies have discovered a correlation between the consumption of fish and the occurrence of cardiovascular disease to lower the risk of serious cardiovascular disease by 7% for every 20 g/day of diet fish [104]. Omega-3 fatty acid consumption is crucial because it increases lipoprotein lipase activity, a mechanism by which clearance accelerates lipoproteins with high triglyceride content. Due to these advantages, the fatty acids omega-3 and omega-6, are necessary and should not be missing from the diet, both preventively and for treating cardiovascular disease. An omega-3/omega-6 ratio of 1:22 or 1:3 and omega-6 to be 5–10% of the total caloric intake, recommended by the American Heart Association. From a dietary point of view, fatty fish are the finest providers of omega-3 fatty acids (tuna, mackerel, salmon, trout) and seafood. Following a study conducted at a school in Denmark, a four-year-old who consumed a diet full of omega-3 fatty acids showed increased reading skills and mathematical calculations [105].

Dietary fiber is recommended due to its antihyperlipidemic effect, which would be: increased activity of hepatic LDL cholesterol receptors in the course of the mycelium formation process links food cholesterol, facilitation of LDL cholesterol clearance, inhibition of hepatic fatty acid synthesis, a decrease in macronutrient absorption, but also increased satiety through increased amount of food. This reduces lipid fraction concentrations by between 0 and 18% for oat products and 3–17% for psyllium. Although the influence is modest, due to the overall beneficial effects on diseases, at least 10g/day is recommended for patients with metabolic syndrome [106,107].

## 3. Discussions

According to the information collected after going through the bibliography used, these are some of the primary and easy dietary recommendations that we can use in the prevention of metabolic syndrome, both primary and secondary [108]: Select foods that have had little processing, such as fruits and vegetables, whole grain products, lentils, and lean meats. These type of foods are rich in nutrients and tend to be lower in calories, which can help to reduce weight and improve blood sugar control [75]. Consuming large amounts of added sugars and refined grains can contribute to developing resistance to insulin and overweight or obesity, both of which are hallmarks of metabolic syndrome. Limiting these foods can help to improve blood sugar control and reduce weight [75]. Choose healthy fats like olive oil, almonds, and avocados over harmful ones like trans fats and fats that are saturated. Omega-3 fatty acids possess the ability to reduce cholesterol levels and decrease the probability of developing heart disease [75]. Eat a variety of foods to ensure you consume a range of nutrients. This can benefit general health and lower the chance of acquiring vitamin deficiencies [108]. It is crucial to remember that individual dietary guidelines for metabolic syndrome may vary depending on their individual needs and goals. Working with a registered dietitian or other health care professional to develop a personalized plan is always a good idea. Specific dietary recommendations for individuals with metabolic syndrome may vary depending on their individual needs and goals. Working with a registered dietitian or other health care professional to develop a personalized plan is always a good idea [109]. When implementing a lipid-lowering diet, understanding the patient’s opinion and preferences is essential to facilitate compliance with a diet. Another critical factor is that talking to your family and relatives makes them understand their role and importance, especially for those who procure and cook food [105]. A patient’s lifestyle is essential in controlling metabolic syndrome, especially in those with increased cardiovascular risk or if it is very high. Regarding food intake, replacing fatty acids and trans fats with monounsaturated and polyunsaturated fatty acids is an essential non-pharmacological therapeutic means for controlling serum levels of HDL cholesterol, LDL cholesterol, and total cholesterol [105,110]. Lifestyle optimization is necessary following: an antihypertensive diet, exercise, weight loss, reduced alcohol consumption, smoking cessation, but also psychosocial optimization. Quitting smoking or maintaining a non-smoking status is part of the mandatory recommendations for managing the life of people with metabolic syndrome [105].

## 4. Conclusions

When it comes to protecting against metabolic syndrome, healthy eating habits are more effective than restricting food intake since they include the accumulation of little dietary alterations. When compared to diets that are low in fat and extremely restricted, the Mediterranean diet approach is supported by scientific evidence as an innovative technique for the treatment and therapy of metabolic syndrome. As a result of the Mediterranean diet’s high nutritional diversity and quality, medical professionals are able to provide dietary recommendations that are easy to follow and do not involve a restricted diet. The risk factors for metabolic syndrome can be controlled and general health can be improved with the help of a specialized diet. In light of the fact that the start of cardiovascular disease may be caused by a combination of inherited and environmental variables, personalized dietary interventions may make it easier to investigate novel therapeutic approaches for the purpose of preventing cardiovascular disease and improving overall health. Disease and nutritional deficiencies have typically been the primary areas of concern in the field of nutrition during its development. The impact of metabolic disease can now be investigated through dietetics thanks to recent research on this subject, which has opened up a new route for this investigation.

## Figures and Tables

**Table 1:** The diagnostic criteria for metabolic syndrome have evolved throughout time [15,17].

Clinical Measure	WHO (1998)	NCEP-ATP III (2004)	IDF (2005)	JIS (2009)	Metabolic Syndrome 2022 Diagnostic Criteria
Criteria needed for the definition	Insulin resistance + at least two further measures needed	At least three of the following	Central obesity (WC) + at least two of the following	At least three of the following	Obesity + minimum 2 of 3 additional criteria
Central obesity	Males must have a WHR greater than 0.90, while females must have a WHR greater than 0.85. Additionally, individuals of any gender must have a BMI greater than 30 kg/m^2^.	For males, the waist circumference should be equal to or greater than 102 cm. For females, the waist circumference should be equal to or greater than 88 cm.	For European males, the minimum waist circumference is 94 cm or greater. For European females, the minimum waist circumference is 80 cm or greater.	In European males, the waist circumference should be more than or equal to 94 cm. In European females, the waist circumference should be greater than or equal to 80 cm.	Waist circumference criteria for females is a minimum of 88 cm, while for males it is a minimum of 102 cm. Alternatively, a body mass index (BMI) of 30 kg/m^2^ or higher is also considered.
Lipid metabolism	The TG level should be equal to or greater than 150 mg/dL (1.7 mmol/L), and/or the HDL cholesterol level in males should be less than 35 mg/dL (0.9 mmol/L). In females, HDL-C level should be less than 39 mg/dL (1.0 mmol/L).	The triglyceride level is equal to or more than 150 mg/dL (1.7 mmol/L).	The triglyceride level is greater than or equal to 150 mg/dL (1.7 mmol/L).	The triglyceride level is equal to or more than 150 mg/dL (1.7 mmol/L).	High non-HDL cholesterol level: The non-HDL cholesterol level is equal to or greater than 130 mg/dL, or the individual is currently undergoing therapy with lipid-lowering drugs.
		In males, the HDL-C level is below 40 mg/dL (1.03 mmol/L). In females, the HDL-C level is below 50 mg/dL (1.3 mmol/L).	In men, HDL-C level is below 40 mg/dL (1.03 mmol/L). In females, HDL-C level is below 50 mg/dL (1.3 mmol/L).	In males, the HDL-C level is below 40 mg/dL (1.03 mmol/L). In female the HDL-C level is below 50 mg/dL (1.3 mmol/L).	
Blood pressure (mmHg)	High blood pressure over 140/90 or the individual is taking antihypertensive drugs.	Blood pressure should be ≥130/85.	Blood pressure should be ≥130/85 or the individual is taking antihypertensive medication.	Blood pressure should be ≥130/85 or the individual is taking antihypertensive medication	Systolic blood pressure ≥130 and/or diastolic blood pressure ≥85 mm Hg (control measurement) OR Systolic blood pressure ≥ 130 and/or diastolic blood pressure ≥ 80 mm Hg (home measurement) OR on antihypertensive treatment.
Glucose metabolism/insulin resistance	The FPG level should be equal to or more than 110 mg/dL (6.1 mmol/L) in order to indicate IFG or a diagnosis of T2DM.	The FPG level is greater than 100 mg/dL (5.6 mmol/L) or the individual is taking hyperlipemia medication.	The FPG levels equal to or more than 100 mg/dL (5.6 mmol/L) in order to indicate a diagnosis of T2DM.	Fasting plasma glucose (FPG) levels equal to or more than 100 mg/dL (5.6 mmol/L) or the use of medication.	A fasting glucose level of 100 mg/dL or higher, or a glucose level of 140 mg/dL or higher following a 2-h oral glucose tolerance test, indicates abnormal glucose metabolism. Alternatively, a HbA1c level of 5.7% or higher also indicates abnormal glucose metabolism. Undergoing pharmacotherapy for reducing blood glucose levels.

WHO = World Health Organization, NCEP-ATP III = National Cholesterol Education Program Adult Treatment Panel III, IDF = International Diabetes Federation, JIS = Joint Interim Statement, M = male, F = female, IFG = impaired fasting glucose, FPG = fasting plasma glucose, T2DM = type 2 diabetes mellitus, WC = waist circumference, WHR = waist to hip ratio, BMI = body mass index, TG = triglycerides, and HDL-C = high-density lipoprotein cholesterol.

**Table 2:** Types of diet and the impact on the clinical parameters [98].

Type of Diet	% Distribution of Nutrients from the Daily Caloric Requirements	Improvements in the Clinical Parameters
Mediterranean	35–45% of total fat (mainly monounsaturated fat) 35–45% of carbohydrates 15–18% of proteins	Reduction in cardiovascular disease incidence and outcomes Decreased blood pressure (systolic and diastolic) The inverse association with mortality Improvements in dyslipidemia Decrease in the incidence of type II diabetes
Plant-based diets	Reducing or restricting food of animal origin High consumption of food from vegetable sources Fat repartition rich in unsaturated fats	Reduction in blood pressure (systolic and diastolic) Body weight loss and obesity risk Reducing the risk of cardiovascular diseases Decrease in all-cause mortality Low risk of type II diabetes
Low-fat diet	Total fat <30% kcal/day (<10% from saturated fat) Proteins 15–17% Carbohydrates 50–60%	Reduction in blood pressure (systolic and diastolic) Short-term improvements in cholesterol profile Short-term weight loss Reduced risk of all-cause mortality
DASH (Dietary Approaches to Stop Hypertension)	Total fat 27% Saturated fat 6% Dietary cholesterol Carbohydrates 55% Protein 18%	Reduction in blood pressure (systolic and diastolic) Reduction in BMI and waist circumference Improving the cardiometabolic profile Reducing the incidence of type II diabetes

## Abbreviations

MS	metabolic syndrome
BMI	body mass index
WHO	World Health Organization
IGT	impaired tolerance to glucose
DM	diabetes mellitus
WHR	waist/hip relation
TG	triglyceride
IDF	International Diabetes Federation
CVD	cardiovascular disease
WC	central obesity
LDL	low-density lipoprotein cholesterol
HDL	high-density lipoprotein cholesterol
CRP	C-reactive protein
hsCRP	high-sensitivity CRP
CVE	cardiovascular events
IL-6	interleukin
GA	fatty acids
DHAP	dihydroxyacetone phosphate
acetyl CoA	acetyl coenzyme A
VLDL cholesterol	very low-density lipoprotein cholesterol
MI	myocardial infarction
IGF2	insulin-like growth factor 2
TNF	tumor necrosis factor
NASH	non-alcoholic steatohepatitis
MAFLD	metabolic dysfunction-associated fatty liver illness
NAFLD	non-alcoholic fatty liver disease
HCC	hepatocellular carcinoma
SFA	saturated fatty acids
MUFAs	monounsaturated fats
PUFAs	polyunsaturated
MedDiet	Mediterranean diet
PAI-1	plasminogen activation-1
T2D	type 2 diabetes
NF-kB	nuclear factor kappa B
sVCAM-1	soluble vascular cell adhesion molecule-1
SAA	plasma amyloid A
EPA	eicosapentaenoic acid
DHA	docosahexaenoic acid
IGF-1	insulin-like growth factor 1
DASH	Dietary Approaches to Stop Hypertension
